# Human Milk Microbiota Across Lactation Stages and Free Glutamate Concentrations in Healthy Ecuadorian Women

**DOI:** 10.3390/nu17050805

**Published:** 2025-02-26

**Authors:** Manuel E. Baldeon, Paul Cardenas, Valentina Arevalo, Belen Prado-Vivar, Mario Uchimiya, Lizbeth Peña, Andrea Denisse Benitez, Andrés Suárez-Jaramillo, Arthur S. Edison, Alonso Herrera, Linda Arturo, Marco Fornasini

**Affiliations:** 1 School of Medicine, Universidad Internacional del Ecuador, Quito 170411, Ecuador; jvarevalo.granda@gmail.com (V.A.); mafornasinisa@uide.edu.ec (M.F.); 2 Institute of Microbiology, Universidad San Francisco de Quito, Quito 170901, Ecuador; pacardenas@usfq.edu.ec (P.C.); pradovivar.m@wehi.edu.au (B.P.-V.);; 3 Mueller Lab, Walter and Eliza Hall Institute of Medical Research, Melbourne 3083, Australia; 4 Department of Medical Biology, University of Melbourne, Melbourne 3052, Australia; 5 Complex Carbohydrate Research Center, University of Georgia, Athens, GA 30602, USAaedison@uga.edu (A.S.E.); 6 Hospital Gineco Obstetrico Isidro Ayora, Quito 170136, Ecuador; halonzzin@gmail.com (A.H.); linda.arturo@gmail.com (L.A.)

**Keywords:** human milk, microbiome, free glutamate, lactation stages, breastfeeding, Ecuador

## Abstract

**Background/Objectives:** There is limited information on human milk (HM) microbiome composition and function in Latin America. Also, interactions between HM constituents and its microbiome have received partial attention. Objective: To characterize the HM microbiota composition considering lactation stages (colostrum, transition, and mature HM) and free glutamate concentrations in Ecuadorian mothers. **Methods:** We recruited 20 mothers that gave birth to normal full-term babies and donated colostrum, transition, and mature milk. Samples were assessed by 16S rRNA gene sequencing by Oxford Nanopore Technologies (ONT). Free glutamate concentrations were measured by proton nuclear magnetic resonance (NMR) spectroscopy. **Results:** For each lactation stage and in order of frequency, the majority of ASVs were assigned to *Staphylococcus*, *Streptococcus*, (Firmicutes); *Escherichia*, *Acinetobacter*, (Proteobacteria); *Corynebacterium*, *Lactobacillus*, *Cutibacterium*, (Actinobacteriota); *Chryseobacterium*, and *Flavobacterium* (Bacteroidota). Alfa diversity was similar in HM samples and tended to be higher in milk intended for male infants. We observed significant differences in qualitative β-diversity metrics between samples with low and high glutamate concentrations. Functional predictions of HM microbiota demonstrated the presence of polyamine biosynthesis II super pathway in samples with high glutamate concentrations. **Conclusions:** The core bacterial components of the HM microbiota in Ecuadorian women were similar to those reported from different parts of the world, with variations at the genus level. Free glutamate dynamics in HM need to be studied considering maternal production and bacterial metabolism to better understand HM composition for optimal infant nutrition.

## 1. Introduction

Human milk is a complex biological system, and it is the most important dietary food during early infant development [1]. Human milk’s bioactive composition changes in response to the nutritional and immunological requirements of the lactating infant [2]. Human milk is synthesized, secreted, and delivered by the mammary glands to the lactating infant to ensure optimal nourishment, protection, and development [3]. In addition to the ideal nutritional components of human milk, this biofluid also contains several bioactive factors, including immune and stem cells, interleukins, specific antibodies (IgA, IgG), hormones, defensins, oligosaccharides, peptides, and free amino acids (FAAs), among others [2,4]. Studies have also reported the presence of human milk microbiota, including bacterial species, in the milk of lactating mothers [5]. Although it has been recognized that HM microbiota varies worldwide, the predominant genera have been identified, including *Staphylococcus*, *Streptococcus*, *Pseudomonas*, *Acinetobacter*, *Bifidobacterium*, *Mesorhizobium*, *Brevundimonas*, *Flavobacterium*, and *Rhodococcus*, during the first months of lactation [5,6,7]. Increasing evidence shows the significant effects of HM microbiota on a newborn’s gut colonization and infant development [8,9,10]. However, there is limited information on the potential interaction between HM constituents, including free amino acids, and the composition of the HM microbiome [5,9].

The composition of human milk microbiota is influenced by the mother’s genetics and environmental factors such as geographic location, ethnicity, diet, health status, mode of delivery, term and preterm delivery, and stage of lactation [6,11]. There is limited information on HM microbiome composition in Latin America. Most studies on this important theme have been carried in other regions of the world. Kumar et al. have shown that human milk microbiota from healthy mothers in Europe (Spain and Finland), Africa (South Africa), and Asia (China) differs significantly between countries [12]. Across countries, 23 phylotypes were shared in HM microbiota at the family level. *Lactobacillaceae* were uniquely found in Finnish and *Bifidobactericceae* in South African samples, and *Enterococcaceae* were absent in Chinese HM [12]. Also, among women that delivered vaginally, those in Spain had the highest amounts of Bacteroidetes, while Chinese women had high Actinobacteria [12]. Among all studied women, Spanish and South African women showed significantly higher bacterial genes associated with carbohydrate, lipid, and amino acid metabolism [12]. Another multinational study that included countries in Europe, Africa, Asia, and South America sought to specifically identify the occurrence of *Lactobacillus reuteri*, a natural probiotic in HM, and showed that there are essential differences in the abundance of this species among countries and that only 15% of samples had detectable levels of *L. reuteri* [13]. Human milk composition also varies in response to the stages of lactation and significantly, according to the environment where the mother–infant dyad lives [14]. These data underline the importance of generating information on HM microbiota and its function from regions with different ethnic and cultural characteristics worldwide.

Few studies have analyzed the potential role of HM components and their role in HM microbiome structure and function. Complex biological systems such as HM are rich in macro- and micro-nutrients, including FAAs, that can be used by the microbiota for their maintenance and function [14,15]. The relative abundance of these nutrients could influence the microbiota composition of HM. For instance, the abundance of *Acinetobacter* has been positively associated with HM fat; *Bacillus*, *Peptoniphilus*, and *Anaerococcus* with protein content; and *Enterobacter* and *Actinomyces* with high levels of lactose [15]. Significantly, in a preliminary study, free threonine in HM has been negatively associated with Gammaproteobacteria abundance in the gut, indicating a potential protective role of this FAA against intestinal Enterobacteriaceae for the lactating infant [16]. These results show promoting as well as antagonistic effects of nutrients for HM microbiota composition. Concentrations of free amino acids in HM change with the stages of lactation that parallel changes in HM microbiota composition, particularly of free glutamate, which is the most abundant non-essential amino acid in colostrum, transition, and mature milk [15,17]. Our group and others have shown that FAA concentrations increase with lactation time, with taurine, glutamate, glutamine, and alanine being most abundant in colostrum, transition, and mature milk [18,19]. In addition, we have found significantly higher concentrations of glutamate, glycine, cysteine, and tyrosine in HM intended for males and higher concentrations of glutamate and alanine in HM for children that presented faster weight gain than for those with slower gain [18]. Since concentrations of glutamate increase during lactation, and it is more abundant in HM intended for lactating males, we wanted to characterize the microbiome in colostrum, transition milk, and mature milk in primiparous healthy Ecuadorian women and associate its composition and function with the changes in the abundance of free glutamate in HM intended for males and females born at term.

## 2. Materials and Methods

### 2.1. Study Design

In this cohort study, we assessed the microbiota composition in HM intended for female and male lactating infants. Dyads—mothers and their babies—were recruited at the largest public maternity facility, Hospital Gineco Obstétrico Isidro Ayora in Quito, Ecuador. We recruited permanent resident mothers who indicated their willingness to exclusively breastfeed for at least 2 months. At the time of recruitment, mothers were healthy and had given birth to a full-term baby via vaginal deliveries; mothers were ≥18 years of age (primiparous) and had not consumed antibiotics or probiotics during their pregnancies. A total of 20 mothers donated their colostrum (3.3 ± 0.2 days postpartum, range = 0–7), transition milk (15.1 ± 0.1 days postpartum, range = 13–17), and mature milk (33.4 ± 0.2 30 days postpartum, range = 28–4) [20]. Sociodemographic and clinical characteristics of participating lactating mothers and their children were taken from their hospital clinical records.

### 2.2. Human Milk Sample Collection

The HM collection process was conducted in basal conditions after overnight fasting. Sampling was consistent and intended to prevent contamination [17]. Participating mothers were asked to wash their hands and breasts with warm water and to obtain their milk with a manual sterile pump from both breasts into a sterile container; a range of 7.5 to 20 mL was usually collected. Samples were then transported at approximately 4 °C (refrigeration temperature) to the laboratory within the first 2 h of sampling. Upon arrival, milk samples were processed immediately [17].

### 2.3. Bacterial Load

Bacterial DNA was quantified using the Femto Bacterial DNA Quantification Kit (Zymo Research Corporation, Eugene, OR, USA) according to the manufacturing protocol. Standards were prepared using 18 μL of the Femto Bacterial qPCR Premix and adding 2 μL of Bacterial DNA Standards (#1–7). The kit provides an exact quantity of bacterial DNA present for the standard curve and gives an approximate number of bacterial cells based on *E. coli* strain JM109 copy number. This kit contains a primer mix targeting the 16S rRNA. For samples, 18 uL of Premix was used (1 nM), and 1–3 μL of each sample was added. The qPCR conditions for amplification consisted of initial denaturation or 95 °C × 10 min, 40 cycles of 95 °C × 30 s, 50 °C × 30 s, 72 °C × 1 min, and a final extension of 72 °C × 7 min using the CFX96 Touch Real-Time PCR Detection System (Bio-Rad, Laboratories, Inc., Hercules, CA, USA). Bands were visualized using GelRed (San Francisco, CA, USA) in a 1% agarose gel.

The standard curve was used to quantify the amount of bacterial DNA in the unknown samples for the analysis.

### 2.4. Microbiota Analysis

DNA was extracted from 60 human milk samples using PureLink Genomic DNA Mini Kit (Invitrogen, Carlsbad, CA, USA), following the protocol reported previously [21]. Prior to DNA extraction, samples (4.5 mL) were centrifuged at 15,000× *g* for 3 min at 4 °C, and the fat layer and the supernatant were removed. The DNA quality and quantity were evaluated using the Qubit dsDNA BR assay kit (Invitrogen, Carlsbad, CA, USA), and Nanodrop (Thermo Fisher Scientific, Waltham, MA, USA) was used to measure 260/280 and 260/230 ratios. Subsequently, the complete 16S rRNA gene was amplified using primers 27F and 1492R [22] and the enzyme Q5 High-Fidelity 2X Master Mix (New England Biolabs Inc., Ipswich, MA, USA). The PCR conditions for amplification consisted of initial denaturation at 98 °C × 2 min, 35 cycles of 98 °C × 30 s, 45 °C × 60 s, 72 °C × 2 min, followed by a final extension at 72 °C × 7 min and holding at 8 °C using the CFX96 Touch Real-Time PCR Detection System (Bio-Rad, Laboratories, Inc., Hercules, USA). PCR products were visualized to confirm amplification using gel electrophoresis (1X TBE, 1% agarose, 100 V).

### 2.5. Library Preparation and Sequencing

16S rRNA sequencing of the 60 PCR amplicons was performed following the manufacturer’s instructions (Oxford Nanopore Technologies-ONT, Oxford, UK) using the Native Barcoding kit 24 (SQK-NBD114.24, Oxford Nanopore Technologies-ONT, UK). The genomic library was loaded into a flow cell (FLO-MIN114) and loaded into a GridION device. Sequencing was run in MinKNOW v23.11.7 Software, with super accuracy base calling, barcoding, and adapter removal enabled.

### 2.6. Microbiota Data Analysis

All the data collected during the study (metadata) were validated using the *Keemei* add-on in Google Drive to perform the microbiome analysis [23]. For bacteria taxonomic analysis, the entire MetONTIME meta-barcoding *pipeline from fastq.gz files* was used [24]. The reads were dereplicated using *qiime search dereplicate-sequences*, and then the representative sequences (rep-seqs) and feature table were obtained using the *qiime search cluster-features-de-novo*. The rep-seqs were mapped with Greengenes2 database version 2022.10 to obtain the taxonomic classification, which integrates genomic and 16S rRNA databases into a unified reference tree [25].

The amplicon sequence variants (ASVs) were imported into QIIME2 Software (version qiime2-2023.5) to estimate relative abundance and perform microbial diversity analysis; subsequently, singletons were removed. First, the representative sequences were aligned using the MAFFT algorithm. A phylogenetic tree was then constructed from the aligned sequences using the *qiime phylogeny align-to-tree-mafft-fasttree* plugin to facilitate subsequent diversity unifrac analysis. Diversity analyses were performed using the *q2-diversity plugin*. Alpha diversity calculations (the number of bacterial species per sample) were estimated by the Shannon index (quantitative community richness), observed OTUs (qualitative community richness), Faith’s Phylogenetic Diversity (qualitative community richness with phylogenetic relationships), and Pielou’s evenness (to evaluate equitability). In addition, to assess β-diversity among groups of samples (differences in the complete microbiota patterns), Jaccard metric distance (qualitative community dissimilarity), Bray–Curtis distance (quantitative community dissimilarity), unweighted UniFrac distance (qualitative community with phylogenetic relationships), and weighted UniFrac distance (quantitative community dissimilarity with phylogenetic relationships) were performed [26]. All the calculations were compared between the three lactation periods; also, to assess the potential influence of glutamate concentrations on microbiota composition, samples were divided into three concentrations of glutamate following tertile distribution (low, medium, and high; see free glutamate metabolic analysis section).

To assess changes between paired samples from two different “lactation stages” or glutamate concentrations, pairwise comparisons were performed using the *q2-longitudinal* plugin [27], where the gender of the children was compared between 2 times of collection of HM samples or 2 glutamate concentrations. Finally, the frequency of 20 important features across the three groups studied was computed using the *qiime longitudinal maturity-index command*, with the --p-stratify parameter [27].

### 2.7. Prediction of Functional Pathways in Microbiota Data

Phylogenetic Investigation of Communities by Reconstruction of Unobserved States (PICRUSt2) v2.5.2 [28] was used to infer the functional profile based on 16S rRNA gene sequences. As a result, MetaCyc pathway abundances were predicted. Pathway differential expression was analyzed with Linear discriminant analysis Effect Size (LEfSe) [29]. The threshold on the linear discriminant score (LDA) for discriminative pathways was set to ≥2.0. Colostrum, transition, and mature HM samples and HM samples with low, medium, and high free glutamate concentrations were analyzed.

### 2.8. Free Glutamate Analysis

For extracting polar metabolites, a modified Folch method was used [30]. Two milliliters of chloroform-methanol (2:1) was added to 400 μL of each sample in a 1.5 mL glass vial. After extraction, the polar phase was dried in a speed vacuum centrifuge SC210A SpeedVac Plus (Thermo Savant, USA) overnight. To reconstitute the polar phase, 700 μL of D_2_O phosphate buffer (pH 7.4) with 1M of 3-trimethylsilyl propionic acid (TSP) was added to the dried extract. Then, 700 μL of the reconstituted polar phase was transferred to 5 mm NMR tubes (Bruker) for NMR analysis.

HNMR spectra were collected using a Bruker Avance III HD 600 NMR spectrometer equipped with a 5 mm TCI cryoprobe at 298 K. ^1^H-detection *J*-resolved experiment jresgpprqf (Bruker, Rheinstetten, Germany) was used for spectrum collection with number of scans of 4, spectral width of 16.6 ppm, and number of points of 32 k and 60 for f2 and f1, respectively. TopSpin version 3.6.4 (Bruker) was used for operation.

NMR spectra were processed by NMRPipe [31], and the data were further analyzed using metabolomics-toolbox (https://github.com/edisonomics/metabolomics_toolbox, accessed on 11 January 2025) on MATLAB (R2024b, MathWorks, Natick, MA, USA). JRES spectra were segmented (function, ‘segment 2D’), and peak intensity was extracted (‘bin2D’) and normalized (‘normalize’) by Probable Quotient Normalization [32]. Glutamate was annotated based on a spiking experiment. All the raw NMR data, NMRPipe processing scripts, and a MATLAB workflow script are deposited to Metabolomics Workbench with Study ID ST003363.

All human milk samples collected were divided into three concentrations of glutamate following tertile distribution.

### 2.9. Statistical Analysis

Descriptive statistics were used for all the data collected. SPSS (v. 28.0, IBM Corp., Armonk, NY, USA) software was used for data analysis. To compare the taxonomic composition in beta diversity analysis with the categorical data collected in the study, we used a non-parametric multivariate statistical permutation test: Permutational analysis of variance (PERMANOVA). Additionally, Kruskal–Wallis was used to determine the statistical significance of alpha diversity with the categorical data. *p* values ≤ 0.05 were statistically significant.

The data presented in this study are openly available in https://github.com/marcovfornasini/human_milk_microbiota.git, accessed on 11 January 2025; reference number [63705045].

## 3. Results

### 3.1. Clinical and Sociodemographic Characteristics of the Study Population

Table 1 shows the sociodemographic characteristics of participating lactating mothers. All of the women were adults. Most were housewives, mestizo (someone of Spanish and indigenous descent), with high school education, and half were single. All the participants gave birth to full-term babies through vaginal delivery and exclusively breastfed their children during the study period.

The nutritional characteristics of participating children are shown in Table 2. At the time of delivery, based on weight to height ratio, most children were well nourished, two children were undernourished (one girl, one boy), and two presented overweight and obesity, Table 2. There were no statistically significant differences between males and females in their nutritional parameters. Concerning the head circumferences, the risk of presenting microcephaly was frequent among participating children, and four presented macrocephaly according to the WHO tables-z-scores. There were no statistical differences in head circumferences between males and females in the study population.

### 3.2. Bacterial Load in Colostrum, Transition, and Mature Milk

**Figure 1:**
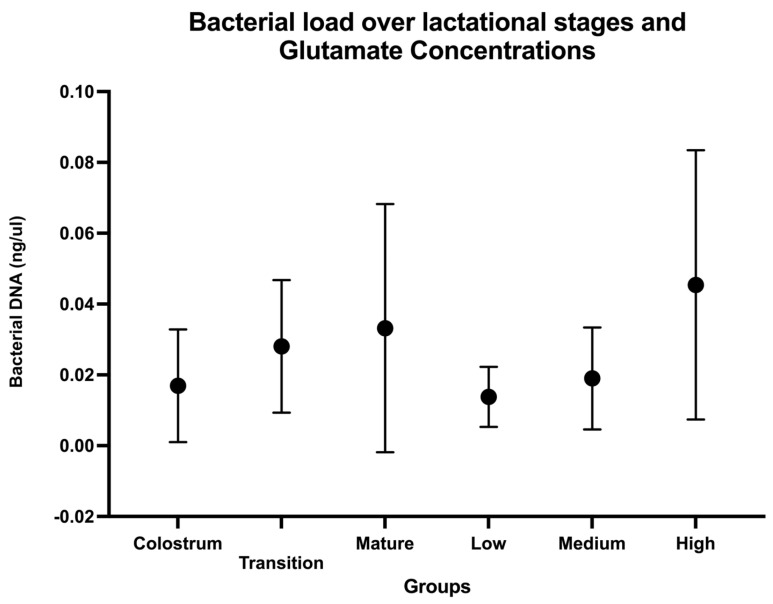
Bacterial DNA concentrations over lactation stages and free glutamate concentrations in human milk samples. Median bacterial DNA from 16S rRNA gene with ranges (maximum and minimum values) for colostrum, transition, and mature human milk and in samples with low, medium, and high glutamate concentrations.

Figure 1 shows bacterial DNA concentrations in HM samples at each lactation stage and three free glutamate concentrations. Mean DNA concentrations increased from colostrum to transition and mature HM. Bacterial DNA was also higher in HM samples with high glutamate concentrations than in samples with medium and low concentrations. There were large variations in bacterial DNA content in samples, indicating that bacterial content was highly variable, Figure 1. These large variations in bacterial DNA concentrations prevented us from establishing significant statistical differences in these samples.

### 3.3. Microbiota Analysis

After the microbiota analysis of the 16S rRNA gene and singleton removal, 766 ASVs were identified. An average of 8593.8 sequences per sample were analyzed. The sequencing experiment produced a range of approximately 5000 to 11,000 reads per sample.

**Figure 2:**
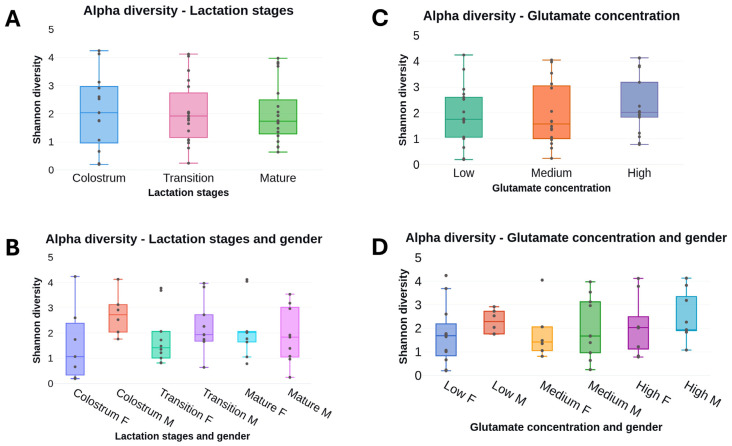
Alpha diversity analysis of observed amplicon sequence variants (ASVs) considering lactation stages and free glutamate concentrations in human milk samples. α-diversity calculations (the number of bacterial species per sample) were estimated by the Shannon index, Faith’s Phylogenetic Diversity, and Pielou’s evenness. (**A**) Shannon index alpha diversity comparison of amplicon sequence variants (ASVs) between lactation stages; and (**C**) three concentrations of free glutamate in human milk samples. (**B**) Alpha diversity comparison between lactation stages in human milk intended for female and male infants; and (**D**) between three free glutamate concentrations in milk for female and male children. F: female; M: male.

Initially, microbiota diversity was assessed considering samples from each lactation stage, namely, colostrum, transition, and mature HM. Alpha diversity was similar in the microbiota of the three lactation stages within the first month of lactation (Shannon index, Faith’s Phylogenetic Diversity, and evenness index), with a more constrained distribution of Shannon values as lactation progressed, Figure 2. Considering the sex of the lactating children, α-diversity was higher in HM intended for males than in HM for females, although those differences were not statistically significant, Figure 2 and Supplementary Materials Table S1. Differences in α-diversity in HM intended for females and males decreased with the time of lactation, additional Supplementary Materials Table S1.

Free glutamate concentrations increased during the three lactation stages, and the medium values with their interquartile range were as follows: colostrum (0.00707 ± 0.00476), transition (0.01192 ± 0.00691), and mature human milk (0.01505 ± 0.00600). To assess HM microbiota diversity considering the concentrations of free glutamate, collected samples were divided into three concentrations of glutamate following tertile distribution: low (range 0.00262 to 0.00805), intermediate (0.00847–0.01361), and high (0.01376–0.09095). Similarly to the observations considering the lactation stages, α-diversity was not significantly different in HM samples with low, intermediate, and high glutamate concentrations (Shannon and Faith’s Phylogenetic Diversity, evenness indices) Figure 2 and Supplementary Materials Table S2. However, mean α-diversity was higher in HM samples with high glutamate concentrations. Also, considering the children’s sex, α-diversity was greater in HM intended for males than for females in samples with low and medium glutamate concentrations, although those differences were not statistically significant, Figure 2.

Regarding β-diversity, comparisons of microbiota differences in colostrum, transition, and mature HM did not show significant differences between samples, Table 3; in addition, considering the children’s sex, β-diversity was not different in HM samples intended for females and males, Supplementary Materials Table S3.

A similar analysis considering free glutamate concentrations showed that qualitative β-diversity metrics of HM samples with low and high glutamate concentrations were different (Jaccard distance *p* = 0.025; unweighted UniFrac distance *p* = 0.076), Table 4. Considering the children’s sex, β-diversity was not different in HM intended for males compared to that for females in samples with varying glutamate concentrations, additional Supplementary Materials Table S4.

#### Taxonomic Identification of Microbiota

**Figure 3:**
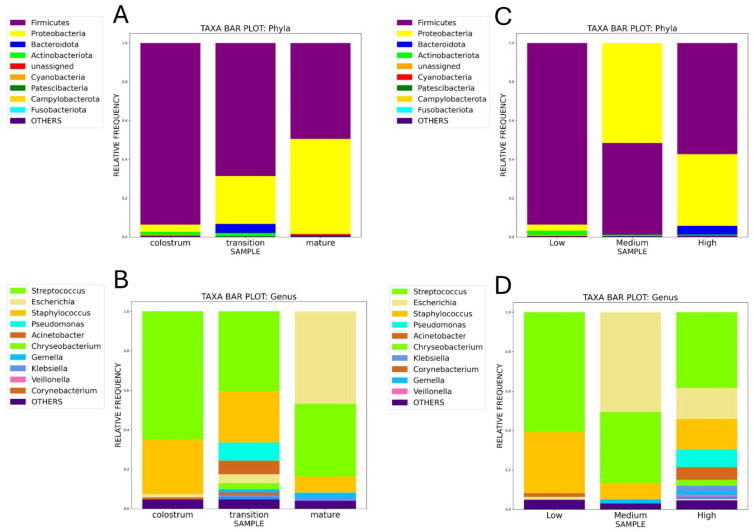
Human milk bacterial microbiota composition. The graphs show the proportions of bacterial phyla (top) and general (bottom) amplicon sequence variants (ASVs) of the 16S rRNA gene. (**A**,**B**) Bacterial composition in colostrum, transition, and mature human milk. (**C**,**D**) Bacterial composition in low, medium, and high free glutamate concentrations. The amplicon sequence variants (ASVs) were imported into QIIME2 Software (version qiime2-2023.5) to estimate relative abundance and perform microbial diversity analysis. The representative sequences were aligned using the MAFFT algorithm.

Table 5 and Table 6 shows the bacterial taxonomic classification at the levels of phylum and genus present in colostrum, transition, and mature milk and at low, medium, and high free glutamate concentrations in HM samples. The representative sequences of the ASVs that were classified using the Greengenes2 database showed that four main phyla were present; in order of frequency, *Firmicutes*, *Proteobacteria*, *Actinobacteriota*, and *Bacteroidota* were more abundantly present in colostrum, transition, and mature milk. Also, a low percentage, 0.93%, of ASVs in all the samples were not assigned to any phylum. There was a trend of decreased abundance of *Firmicutes* (93.6% vs. 75.7%) and *Actinobacteriota* (1.5% vs. 1.38%) in colostrum samples compared to mature HM samples, Figure 3. Contrarily, we observed an increase in the abundance of *Proteobacteria* and *Bacteroidota* in the indicated samples (Proteobacteria, 3.72% vs. 19.64%; *Bacteroidota*, 0.04% vs. 3.11%), Figure 3. In addition, when the microbiota was analyzed pondering the concentrations of free glutamate, Firmicutes, Proteobacteria, Actinobacteriota, and Bacteroidota were present in descending order of frequency, Table 6. Considering glutamate concentrations, we also observed a trend of decreased abundance of Firmicutes (93.82% vs. 55.61%) and Actinobacteriota (2.13% vs. 0.57%) in MH samples with low compared to high glutamate concentrations, Figure 3. Contrarily, we observed an increase in the abundance of Proteobacteria and Bacteroidota at these two glutamate concentrations (Proteobacteria, 3.09% vs. 39.68%; Bacteroidota, 0.04% vs. 3.39%), Table 6. The four main phyla present in colostrum, transition, and mature milk and at low, medium, and high free glutamate concentrations were different in HM samples intended for male and female lactating children, Table 5 and Table 6.

At the genus level for each lactation stage, the majority of ASVs were assigned to *Staphylococcus*, *Streptococcus*, *Escherichia*, *Acinetobacter*, *Corynebacterium*, *Cutibacterium*, *Chryseobacterium*, and *Flavobacterium* taxa, Figure 3B, Table 5. As expected, the genera in the human milk microbiota followed the same pattern as the phyla to which they belonged. Thus, when the percentage of abundance between colostrum and mature milk was compared, the genera of *staphylococcus* (28.87% vs. 19.35%) and *streptococcus* (63.71% vs. 53.47%) decreased. Likewise, the percentages of the genera *Corynebacterium* (1.04 vs. 0.91) and *Cutibacterium* (0.17 vs. 0.12) in colostrum were lower than their respective percentages in mature milk. Contrarily, the percentages of the genus *Escherichia* (1.71% vs. 4.89%) and *Acinetobacter* (0.86 vs. 4.61%) increased from colostrum to mature milk. We observed a similar distribution at the genus level when HM samples were analyzed considering the concentrations of free glutamate. When we compared the percentage of abundance between HM samples with low and high free glutamate concentrations, the genera of *staphylococcus* (34.09% vs. 14.17%) and *streptococcus* (57.89% vs. 37.65%) decreased. Likewise, the percentages of the genera *Corynebacterium* (1.76 vs. 0.14) and *Cutibacterium* (0.14 vs. 0.11) in HM samples with low glutamate concentrations were lower than their respective percentages in HM samples with high glutamate. Contrarily, the percentages of the genus *Escherichia* (1.57% vs. 21.7%) and *Acinetobacter* (0.61 vs. 4.89%) were higher in MH samples with high glutamate concentration than in samples with low concentrations, Figure 3, Table 6. We also observed important variations in bacterial genera of the HM microbiota considering the lactation stages and free glutamate concentration in HM intended for females and males, Figure 3.

**Figure 4:**
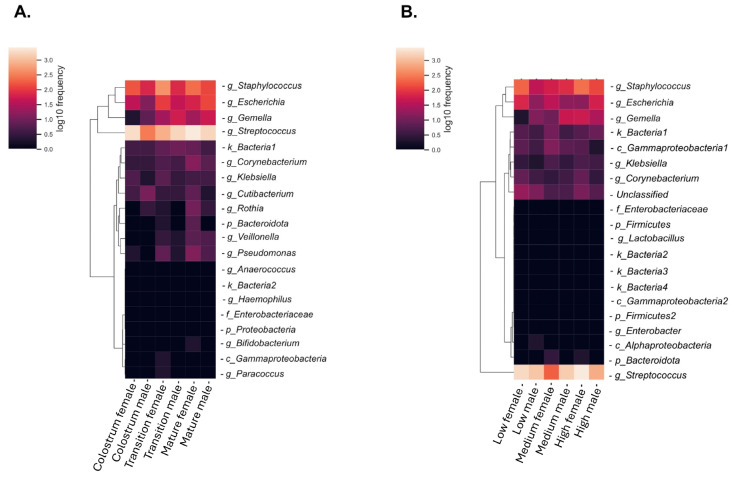
Human milk microbiota associated with (**A**) lactation stages and (**B**) free glutamate concentrations in milk intended for female and male infants. Heat maps based on 20 most abundant genera. Values range from low (black) to high (orange).

The heatmap of the 20 most abundant taxonomic groups in HM demonstrated important microbiota changes during the three lactation periods, Figure 4. In general, we observed fluctuations in bacterial abundance from colostrum to transition and mature HM; some taxa increased, and others decreased with the time of lactation. The observed changes were more obvious with the most abundant taxa, such as *Streptococcus*, *Staphylococcus*, *Escherichia*, and *Gemella*, Figure 4A. A similar pattern of taxon variation was observed in HM with different glutamate concentrations, Figure 4.

### 3.4. Predicted HM Microbiota Functional Profiles During Lactation

To obtain functional predictions based on 16S rRNA, we used PICRUSt2 [28] and LEfSe [29] tools for the analysis. When lactation stages were considered, no pathways had a linear discriminant score (LDA) ≥ 2.0. However, when glutamate concentrations in HM were considered, two MetaCyc pathways had an LDA score ≥ 2.0: (S)-propane-1,2-diol degradation and the super pathway of polyamine biosynthesis II, Supplementary Materials Figure S1. These pathways had greater expression in HM samples with high glutamate concentrations.

## 4. Discussion

The current prospective study described for the first time the structure and function of the human milk microbiota in healthy adult primiparous Ecuadorian lactating women with normal full-term pregnancies who gave birth to healthy children through vaginal deliveries and were exclusively breastfeeding. Alfa diversity was similar in colostrum, transition, and mature HM during the first month of lactation; also, α-diversity tended to be higher in HM intended for male infants. Similar results were observed in HM samples with low, medium, and high glutamate concentrations. However, a comparison of qualitative β-diversity metrics of HM samples with low and high glutamate concentrations showed significant differences between these samples. For each lactation stage, and in order of frequency, the majority of ASVs were assigned to *Staphylococcus*, *Streptococcus*, *Escherichia*, *Acinetobacter*, *Corynebacterium*, *Lactobacillus*, *Cutibacterium*, *Chryseobacterium*, and *Flavobacterium* genera. These bacterial distributions were also present in HM samples with low, medium, and high glutamate concentrations. Functional predictions on HM microbiota based on 16s rRNA gene sequencing and glutamate concentrations demonstrated the presence of the super pathway of polyamine biosynthesis II, which is an essential pathway for lactic acid bacteria (LAB) present in HM with high glutamate concentrations.

There is limited information on the composition of HM in Latin America, particularly concerning the structure and function of the microbiota under physiological conditions. In this study, we analyzed HM microbiota from healthy Ecuadorian lactating women under physiological conditions who gave birth to newborns with normal nutritional status. The process of HM collection was conducted in basal conditions after overnight fasting; sampling was consistent and intended to prevent contamination. Importantly, HM samples were processed within the first 2 h of collection since sample storage affected the yield and quality of isolated DNA. Human milk analysis under physiological conditions is important because these data could be helpful when compared with those of nursing mothers who suffer from obstetric or other diseases such as gestational diabetes, pre-eclampsia, and infections that are common pathologies in Latin America [33,34]. In addition, local studies of HM from normal individuals are significant since its composition is dynamic and greatly changes within the lactating mother and within and among population groups worldwide [14]. It will be necessary to study HM microbiota further under normal or pathological conditions in different regions within Latin America.

Since our study focused on the bacterial characteristics of HM microbiota, it was essential to measure the bacterial load in these samples. For this purpose, the exact quantity of bacterial DNA in colostrum, transition, and mature milk, as well as in HM samples with different glutamate concentrations, was assessed. Despite the large variations in bacterial DNA concentrations, the average DNA in colostrum, transitional, and mature milk tended to increase during the first 4 weeks of breastfeeding studied. Furthermore, bacterial DNA concentrations showed a similar trend in HM samples with increasing glutamate concentrations, indicating a potential relationship between bacterial load and free glutamate in HM. It will be important to study free glutamate dynamics in HM considering maternal glutamate production and bacterial metabolism of this amino acid. There are few reports on bacterial load in HM; an estimation of bacteria-specific DNA by qPCR targeting the *fusA* gene shows wide variability in the number of bacteria in HM samples; the authors reported no significant differences in median values in colostrum, transition, and mature HM, although the average microbial DNA was higher in mature milk than in colostrum. This is similar to the current report [15]. It is possible that the large variations in bacterial DNA and the limited number of HM samples analyzed limited the establishment of significant statistical differences in these samples, but the biological significance of these increments needs to be further addressed.

Once we established the bacterial load, it was important to analyze their diversity in these samples since bacterial diversity is important for maintaining a healthy mucosa [35]. Several studies have analyzed the changes in HM microbiota diversity during lactation, showing conflicting results. A study from three cities in China that included 117 mothers that analyzed their HM at 1, 2, and 6 weeks postpartum indicated that microbial diversity and richness decreased during lactation [36]. A similar study from Ireland that included 80 women and analyzed their HM at 1, 4, 8, and 24 weeks postpartum also reported a decrease in HM microbiota diversity throughout the first 6 months of lactation, with a greater difference between 8 and 24 weeks [7]. Contrarily, a study from The Netherlands that collected HM at 2, 6, and 12 weeks postpartum from 77 lactating women reported an increase in bacterial diversity during the first 3 months of lactation [37]; a similar increase in bacterial diversity during lactation has also been reported in healthy Spanish mothers [38]. Here, in terms of α-diversity, and similarly to previous observations, HM microbiota was similar in colostrum, transition, and mature milk [15]. Comparing microbial richness and evenness in milk intended for males and females, there was a trend of greater bacterial diversity in milk for males than for females in colostrum, transition, and mature HM, and a similar trend was observed in samples with low, medium, and high glutamate concentrations. In this regard, previous work shows that free glutamate in HM increased with lactation time, and its concentration was significantly higher in milk intended for males who presented faster weight gain [17,18]. It has been proposed that HM microbiome composition differs according to the sex of the breastfeeding child, which could be due to the contribution of the breastfeeding child to the bacterial load; this could be related to the retrograde inoculation of different bacteria from the male or female oral cavity at the moment of direct lactation [35,39]. However, other studies have not found differences in the microbiome in milk for male and female lactating children although HM analysis has been carried out at different time points and conditions of the lactation period [7,40]. Further studies including larger groups of lactating mothers are needed to determine the role of infants’ sex on HM microbiota [41].

Comparison of microbiota between colostrum, transition, and mature HM did not show significant differences between HM samples. However, the same analysis considering the concentrations of free glutamate showed that the qualitative β-diversity metrics of HM microbiota were different. These results indicated that the low and high concentrations of glutamate could influence the composition of HM microbiota. Previous work has shown that free amino acids in human milk modulate infants’ intestinal microbiota. For instance, threonine is negatively associated with Gammaproteobacteria and Enterobacteriaceae members [16]. It is possible that free glutamate and other free amino acids could modulate HM microbiota during lactation. Due to the changing nature of nutrients in breast milk, the composition of its microbiota has been associated with those changes during lactation [39]. Based on these observations, it will be important to determine possible mechanisms by which free amino acids and other milk components affect HM and gut microbiota. It is known that LAB identified in this study in samples with high glutamate concentrations, such as *Lactobacillus* and *Streptococcus*, primarily generate energy by lactic fermentation, arginine deamination, and amino acid decarboxylation present in HM [42]. In addition, HM stimulates *Lactobacillus* proliferation and generates several metabolites that are products of the glycosylate cycle (succinate), urea cycle (citrulline), and polyamine synthesis (spermidine) metabolic pathways [43]. Of note, in the present study, the super pathway of polyamine biosynthesis II had a linear discriminant score (LDA) ≥ 2.0 in HM samples with high glutamate concentrations. Polyamines are related to cell proliferation, cell differentiation, protein synthesis, adipogenesis, apoptosis, intestinal maturation, stabilization of negative charges of DNA, RNA transcription, regulation of potassium channels, and immune system development, which could affect infants ‘growth [44,45]. In addition, LAB characteristics enable these bacteria to survive and colonize mucosal environments at the local level and with the host modulating smooth muscle and central nervous system functions [42,46].

Previous work on HM microbiomes from different parts of the world has demonstrated the presence of what has been called a “core” of bacteria consisting of Firmicutes, Proteobacteria, and Actinobacteria at the phylum level (reviewed in [35]). In accordance with those observations, we identified, in order of frequency, Firmicutes, Proteobacteria, Actinobacteriota, and Bacteroidota phyla in percentages of abundance that differ from previous reports [35,38]. In addition, in our study, the abundance of Firmicutes decreased during lactation time, while Proteobacteria showed the opposite trend, and this phenomenon was more evident when we considered the distribution of phyla in samples with different glutamate concentrations. These changes in the phyla were accompanied by changes in the most abundant genera within these phyla: for Firmicutes, changes in *Streptococcus* and *Staphylococcus*, and for Proteobacteria, in *Escherichia* and *Acinetobacter*. As expected, at the genus level, we identified microbial groups similar to those that have been reported previously, such as *Streptococcus*, *Staphylococcus*, *Gemella*, *Enterococcus*, *Pseudomonas*, *Lactobacillus*, *Flavobacterium*, *Acitenobacter*, and *Veillonella* [47,48,49]. Also, a small percentage of the genera *Dolosigranulum*, *Paracoccus*, and *Rahnella*, usually present in the environment or associated with milk, as well as *Pantoea* and *Brevundimonas*, were identified [50]. However, bacteria previously identified as part of the HM microbiota, such as *Clostridium*, *Prevotela*, *Weissella*, *Leuconostoc*, and *Moraxellaceae*, were not found in HM from Ecuadorian lactating mothers [51,52]. It is possible that the inherent characteristics of the Ecuadorian mothers, their physiological conditions, the geographic location of the study, sampling collection and immediate analysis, information on pre/probiotic consumption, delivery mode, and breastfeeding practices could explain the differences in the microbial composition of HM observed in this study compared with previous reports [5]. Together, these data support the notion of echo-homeorhesis for the composition of the normal microbiome in HM. Echo-homeorhesis considers particular adjustments of homeostasis in HM to favor the health of the lactating infant in a specific environment, culture, and geographic location [14].

More specific bacterial taxonomic identification at the species level can further clarify the particularities of the HM microbiome in different populations. Together, these data indicate that although HM bacterial communities vary geographically, they consistently contain core genera that include *Staphylococcus* and *Streptococcus*. These two genera are universally present in HM, regardless of the demographics and geographical location [53,54]. These data also underline the importance of generating local information on HM microbiota.

### Strengths and Limitations

The analysis of HM microbiota was carried out under the physiological conditions of lactating mothers. However, we did not record data on their diet or physical activity, and most of them came from urban areas that could affect HM composition [55,56]. Also, the present study included a limited number of mothers. It is possible that the collection of HM samples could have influenced glutamate levels since we did not collect the totality of milk from the breast. On the other hand, the sequencing methodology used here allowed us to obtain the complete 16sRNA gene sequences, giving more accuracy for identifying bacteria at the genus level in HM intended for male and female infants. Finally, we did not assess other important factors such as human milk oligosaccharides that influence HM microbiota composition.

## 5. Conclusions

The current prospective study described for the first time the structure and function of the human milk microbiota in healthy adult primiparous Ecuadorian lactating women with normal full-term pregnancies who gave birth to healthy children through vaginal deliveries and were exclusively breastfeeding. A comparison of qualitative β-diversity metrics of HM samples with low and high glutamate concentrations showed significant differences between these samples. These results indicate a *potential role* of glutamate in human milk microbiota composition that needs to be further addressed. The core bacterial components of the HM microbiota were like those in previous reports from different parts of the world but presented some variations at the genus level. Functional predictions of HM microbiota demonstrated the presence of the super pathway of polyamine biosynthesis II, an essential pathway for LAB present in HM with high glutamate concentrations. More research is needed to assess free glutamate dynamics in HM considering maternal glutamate production and bacterial metabolism of this important amino acid.

## Figures and Tables

**Table 1:** Sociodemographic characteristics of participating lactating mothers.

	Number (*n* = 20)	Percentage
mean age ± SD	26.7 ± 7.1	
**Occupation**		
Housewife	9	45%
Employee	7	35%
Student	4	20%
**Education**		
Elementary	3	15%
High school	12	60%
University	5	25%
**Marital status**		
Married	6	30%
Divorced	1	5%
Single	9	45%
Civil partnership	4	20%
**Ethnicity**		
Black	1	5%
Indigenous	2	10%
Mestizo	17	85%

**Table 2:** Nutritional characteristics of participating children.

**Breastfed Females (*n* = 10)**				**Breastfed Males (*n* = 10)**			
**Weight to Height Ratio—z-Score** ^1^							
**<−2 Undernourishment (*n*)**	**≥−2/≤1 Normal (*n*)**	**>1/≤2 Overweight (*n*)**	**>2 Obesity (*n*)**	**<−2 Undernourishment (*n*)**	**≥−2/≤1 z Normal (*n*)**	**>1/≤2 Overweight (*n*)**	**>2 Obesity (*n*)**
1	8	1	0	1	8	0	1
**Breastfed Girls (*n* = 9)** ^2^				**Breastfed Boys (*n* = 9)**			
**Head Circumference**							
**<−2 Microcephaly (*n*)**	**≥−2/≤1 Risk of Microcephaly % (*n*)**	**>1/≤2 Normal (*n*)**	**>2 Macrocephaly (*n*)**	**<−2 Microcephaly (*n*)**	**≥−2/≤1 Risk of microcephaly % (*n*)**	**>1/≤2 Normal (*n*)**	**>2 Macrocephaly (*n*)**
0	3	4	2	0	4	3	2

^1^ Anthropometric data were analyzed using Anthro Plus WHO charts: weight-for-length/height for age and head circumference for age. ^2^ Head circumference was not recorded for two children in their clinical records.

**Table 3:** Beta diversity measures across colostrum, transition, and mature human milk samples.

β-Diversity ^1^	Bray–Curtis Distance		Jaccard		Unweighted UniFrac Distance		Weighted UniFrac Distance	
**Lactation stages**	Pseudo-F	*p*-value	Pseudo-F	*p*-value	Pseudo-F	*p*-value	Pseudo-F	*p*-value
**All lactation stages**	0.501	0.910	1.172	0.081	1.086	0.262	0.181	0.997
**Colostrum vs. Transition**	0.489	0.836	1.176	0.160	1.074	0.294	0.126	0.987
**Colostrum vs. Mature**	0.529	0.771	1.089	0.247	1.073	0.306	0.147	0.964
**Transition vs. Mature**	0.484	0.797	1.243	0.095	1.106	0.306	0.258	0.885

^1^ β-diversity between groups of samples was assessed (differences in the complete microbiota patterns), by Jaccard metric distance, Bray–Curtis distance, unweighted UniFrac distance, and weighted UniFrac distance. All the calculations were compared between the three lactation periods.

**Table 4:** Beta diversity measures across human milk samples with different free glutamate concentrations.

β-Diversity ^1^	Bray–Curtis Distance		Jaccard		Unweighted UniFrac Distance		Weighted UniFrac Distance	
**Glutamate concentrations**	Pseudo-F	*p*-value	Pseudo-F	*p*-value	Pseudo-F	*p*-value	Pseudo-F	*p*-value
**All glutamate concentrations**	0.703	0.707	1.154	0.103	1.036	0.381	0.478	0.858
**Low vs. Medium**	0.843	0.455	1.061	0.310	0.813	0.783	0.486	0.720
**Low vs. High**	1.037	0.380	1.411	0.025	1.363	0.076	0.732	0.510
**Medium vs. High**	0.146	0.992	0.968	0.520	0.924	0.586	0.211	0.918

^1^ β-diversity between groups of samples was assessed (differences in the complete microbiota patterns), by Jaccard metric distance, Bray–Curtis distance, unweighted UniFrac distance, and weighted UniFrac distance. All the calculations were compared between HM samples with the three free glutamate concentration.

**Table 5:** Human milk microbiota taxonomic composition by gender and lactation stage.

		Lactation Period					
		Colostrum		Transition Milk		Mature Milk	
	Phylum/*Genus*	HM for Girls	HM for Boys	HM for Girls	HM for Boys	HM for Girls	HM for Boys
**No.**	**Firmicutes**	93.21%	94.14%	44.18%	60.67%	61.87%	89.17%
**1**	*Staphylococcus*	5.70%	52.04%	7.23%	10.11%	32.69%	6.01%
**2**	*Streptococcus*	87.39%	40.03%	33.66%	43.95%	27.49%	79.44%
**3**	*Anaerococcus*	0.05%	0.08%	0.01%	0.01%	0.01%	0.00%
**4**	*Gemella*	0.02%	1.49%	1.91%	3.81%	1.15%	3.22%
**5**	*Veillonella*	0.00%	0.34%	1.09%	1.60%	0.20%	0.19%
**6**	*Lactobacillus*	0.00%	0.09%	0.21%	0.33%	0.03%	0.01%
**7**	*Granulicatella*	0.01%	0.00%	0.02%	0.43%	0.00%	0.00%
**8**	*Dolosigranulum*	0.00%	0.00%	0.00%	0.00%	0.17%	0.00%
**Proteobacteria**		4.34%	3.11%	54.44%	37.61%	29.69%	9.56%
**1**	*Escherichia*	1.06%	2.36%	53.73%	32.25%	4.34%	5.44%
**2**	*Acinetobacter*	1.56%	0.17%	0.19%	0.14%	9.05%	0.18%
**3**	*Brevundimonas*	0.24%	0.02%	0.11%	0.01%	0.04%	0.02%
**4**	*Paracoccus*	0.20%	0.02%	0.03%	0.02%	0.01%	0.04%
**5**	*Haemophilus*	0.42%	0.00%	0.08%	0.03%	0.08%	2.81%
**6**	*Pseudomonas*	0.41%	0.07%	0.10%	0.14%	12.21%	0.12%
**7**	*Klebsiella*	0.06%	0.20%	0.07%	4.82%	1.24%	0.58%
**8**	*Aeromonas*	0.26%	0.06%	0.00%	0.01%	0.33%	0.01%
**9**	*Lelliottia*	0.00%	0.01%	0.00%	0.01%	0.53%	0.00%
**10**	*Rahnella*	0.00%	0.01%	0.01%	0.00%	0.80%	0.01%
**11**	*Pantoea*	0.00%	0.00%	0.00%	0.00%	0.38%	0.00%
**Actinobacteriota**		0.67%	2.32%	0.59%	0.62%	1.91%	0.85%
**1**	*Corynebacterium*	0.29%	1.79%	0.20%	0.22%	1.74%	0.08%
**2**	*Cutibacterium*	0.14%	0.20%	0.05%	0.10%	0.09%	0.16%
**3**	*Rothia*	0.12%	0.08%	0.16%	0.07%	0.07%	0.52%
**Bacteroidota**		0.05%	0.03%	0.12%	0.11%	6.15%	0.08%
**1**	*Chryseobacterium*	0.04%	0.01%	0.05%	0.04%	4.06%	0.02%
**2**	*Flavobacterium*	0.01%	0.01%	0.01%	0.01%	1.37%	0.01%
**3**	*Sphingobacterium*	0.00%	0.01%	0.01%	0.01%	0.61%	0.02%
**Others**		1.09%	0.05%	0.08%	0.12%	0.12%	0.24%
**1**	*Cyanobacteria*	1.05%	0.00%	0.02%	0.01%	0.05%	0.02%
**2**	Staskawiczbacteraceae-unassigned-genus	0.04%	0.05%	0.05%	0.10%	0.06%	0.21%
***Unassigned genus***		0.63%	0.34%	0.59%	0.87%	0.26%	0.11%

The amplicon sequence variants (ASVs) were imported into QIIME2 Software (version qiime2-2023.5) to estimate relative abundance and perform microbial diversity analysis. The representative sequences were aligned using the MAFFT algorithm. Bacterial phyla, in bold, are not italicized, while genera are italicized.

**Table 6:** Human milk microbiota taxonomic composition by gender and free glutamate concentrations.

		Free Glutamate Concentration					
		Low		Medium		High	
	Phylum/*Genus*	HM for Girls	HM for Boys	HM for Girls	HM for Boys	HM for Girls	HM for Boys
**No.**	**Firmicutes**	92.87%	94.77%	26.98%	94.54%	60.52%	50.70%
**1**	*Staphylococcus*	19.49%	48.70%	8.45%	7.62%	18.01%	10.34%
**2**	*Streptococcus*	71.85%	43.94%	17.12%	80.29%	39.78%	35.52%
**3**	*Anaerococcus*	0.05%	0.07%	0.00%	0.01%	0.00%	0.00%
**4**	*Gemella*	1.35%	1.35%	0.16%	6.27%	1.78%	1.89%
**5**	*Veillonella*	0.01%	0.28%	1.02%	0.25%	0.59%	1.85%
**6**	*Lactobacillus*	0.00%	0.08%	0.21%	0.02%	0.10%	0.38%
**7**	*Granulicatella*	0.02%	0.22%	0.01%	0.02%	0.01%	0.13%
**8**	*Dolosigranulum*	0.00%	0.00%	0.00%	0.00%	0.18%	0.00%
**Proteobacteria**		3.62%	2.56%	72.08%	4.03%	31.70%	47.66%
**1**	*Escherichia*	1.24%	1.90%	71.09%	3.22%	4.36%	39.04%
**2**	*Acinetobacter*	1.08%	0.15%	0.52%	0.21%	9.58%	0.14%
**3**	*Brevundimonas*	0.17%	0.02%	0.14%	0.02%	0.04%	0.01%
**4**	*Paracoccus*	0.15%	0.02%	0.02%	0.04%	0.01%	0.02%
**5**	*Haemophilus*	0.28%	0.00%	0.02%	0.03%	0.15%	2.06%
**6**	*Pseudomonas*	0.33%	0.04%	0.10%	0.23%	13.25%	0.11%
**7**	*Klebsiella*	0.05%	0.17%	0.10%	0.11%	1.34%	5.89%
**8**	*Aeromonas*	0.18%	0.06%	0.00%	0.01%	0.36%	0.01%
**9**	*Lelliottia*	0.00%	0.00%	0.00%	0.01%	0.58%	0.01%
**10**	*Rahnella*	0.00%	0.01%	0.01%	0.01%	0.87%	0.01%
**Actinobacteriota**		2.16%	2.10%	0.30%	1.03%	0.49%	0.66%
**1**	*Corynebacterium*	1.85%	1.68%	0.08%	0.15%	0.09%	0.19%
**2**	*Cutibacterium*	0.12%	0.15%	0.03%	0.20%	0.10%	0.13%
**3**	*Rothia*	0.09%	0.02%	0.04%	0.61%	0.22%	0.07%
**Bacteroidota**		0.06%	0.02%	0.09%	0.16%	6.72%	0.07%
**1**	*Chryseobacterium*	0.04%	0.01%	0.05%	0.07%	4.41%	0.01%
**2**	*Flavobacterium*	0.01%	0.00%	0.01%	0.01%	1.48%	0.01%
**3**	*Sphingobacterium*	0.00%	0.00%	0.01%	0.02%	0.66%	0.01%
**Others**		0.78%	0.05%	0.10%	0.13%	0.10%	0.20%
**1**	*Cyanobacteria*	0.72%	0.01%	0.03%	0.01%	0.06%	0.01%
**2**	Staskawiczbacteraceae-unassigned-genus	0.06%	0.04%	0.07%	0.12%	0.03%	0.18%
***Unassigned genus***		0.52%	0.50%	0.45%	0.12%	0.46%	0.70%

The amplicon sequence variants (ASVs) were imported into QIIME2 Software (version qiime2-2023.5) to estimate relative abundance and perform microbial diversity analysis. The representative sequences were aligned using the MAFFT algorithm. Bacterial phyla, in bold, are not italicized while genera are italicized.

## Data Availability

The datasets used and/or analyzed during the current study are available from the corresponding author on reasonable request due to ethical reasons.

## Supplementary Materials

The following supporting information can be downloaded at: https://www.mdpi.com/article/10.3390/nu17050805/s1, Table S1. Comparison of Human Milk α-Diversity Measures Across Different Lactation Stages and between samples for female and male infants. Table S2. Comparison of α-Diversity Measures Across human milk samples with different glutamate concentrations for female and male infants. Table S3. Beta-Diversity measures across colostrum, transition, and mature human milk for female and male lactating infants. Table S4. Comparison of β-diversity Measures Across human milk samples with different glutamate concentrations for female and male lactating infants. Figure S1. Linear discriminant analysis (LDA) scores, combined with effect size measurements (LEfSe) computed for differentially abundant predicted functions among three free glutamate concentrations in human milk samples. The length of the bar represents the log10 transformed LDA score. The threshold on the LDA score for discriminative pathways was set to ≥2.0.
