# Supplementary material for: Human Milk Microbiota Across Lactation Stages and Free Glutamate Concentrations in Healthy Ecuadorian Women
Source: Nutrients. 2025 Feb 26;17(5):805. doi: 10.3390/nu17050805 (PMC11902271; doi:10.3390/nu17050805)
Supplement: Supplementary file 1 [file nutrients-17-00805-s001.zip › nutrients-3403640-supplementary.pdf]

Table S1. Comparison of Human Milk α-Diversity Measures Across Different Lactation Stages and between samples for female and male infants.

**α-diversity**	**Shannon index**	**Observed features**	**Faith's phylogenetic diversity**	**Evenness**
	p-value	*p-value*	*p-value*	*p-value*
All lactation stages	0.929	0.645	0.848	0.820
Colostrum: F vs Colostrum: M	0.086	0.174	0.153	0.086
Transition-: F vs Transition-milk: M	0.151	0.970	0.257	0.112
Mature-: F vs Mature-milk: M	0.514	0.487	0.624	0.414

Alpha Diversity analysis of observed amplicon sequence variants (ASVs) considering lactation stages. α-diversity calculations (the number of bacterial species per sample) were estimated by the Shannon index, Faith's Phylogenetic Diversity, and Pielou's evenness. F: female; M: male

Table S2. Comparison of α-Diversity Measures Across human milk samples with different glutamate concentrations for female and male infants.

**α-diversity**	**Shannon index**	**Observed features**	**Faith's phylogenetic diversity**	**Evenness**
	p-value	*p-value*	*p-value*	*p-value*
All glutamate concentrations	0.346	0.550	0.648	0.350
Low: F vs Low: M	0.092	0.348	1.000	0.160
Medium: F vs Medium: M	0.828	0.871	0.448	0.914
High: F vs High: M	0.691	0.965	0.825	0.508

Alpha Diversity analysis of observed amplicon sequence variants (ASVs) considering free glutamate concentrations. α-diversity calculations (the number of bacterial species per sample) were estimated by the Shannon index, Faith's Phylogenetic Diversity, and Pielou's evenness. F: female; M: male

Table S3. Beta-Diversity measures across colostrum, transition, and mature human milk for female and male lactating infants.

**β-diversity**	**Bray Curtis distance**		**Jaccard**		**Unweighted UniFrac distance**		**Weighted UniFrac distance**	
	Pseudo-F	*p-value*	*Pseudo-F*	*p-value*	*Pseudo-F*	*p-value*	*Pseudo-F*	*p-value*
All lactation stages and gender	0.746	0.793	1.099	0.123	1.034	0.354	0.563	0.918

Colostrum: F vs Colostrum: M	1.096	0.350	1.091	0.250	1.282	0.171	1.314	0.230
Transition-: F vs Transition-milk: M	0.368	0.886	0.890	0.712	0.870	0.659	0.226	0.879
Mature-: F vs Mature-milk: M	1.146	0.322	1.159	0.181	0.808	0.781	0.857	0.476

Beta-diversity analysis of observed amplicon sequence variants (ASVs) considering different lactation stages and between samples for female and male infants. These data represent the comparison of β-diversity measures, including pseudo F-statistic and associated p-values, calculated using Bray-Curtis dissimilarity, Jaccard dissimilarity, weighted UniFrac distance, and unweighted UniFrac distance across human milk samples stratified by lactation stages (colostrum, transition, and mature) and gender (male vs. female).

Table S4. Comparison of β-diversity Measures Across human milk samples with different glutamate concentrations for female and male lactating infants.

β-diversity	Bray Curtis distance		Jaccard		Unweighted UniFrac distance		Weighted UniFrac distance	
	Pseudo-F	*p-value*	*Pseudo-F*	*p-value*	*Pseudo-F*	*p-value*	*Pseudo-F*	*p-value*
All glutamate concentrations and gender	0.710	0.829	1.018	0.374	0.927	0.721	0.716	0.738
Low: F vs Low: M	0.601	0.699	1.181	0.123	1.285	0.110	0.235	0.946
Medium: F vs Medium: M	0.609	0.615	0.803	0.892	0.654	0.967	1.160	0.295
High: F vs High: M	1.016	0.405	0.804	0.857	0.602	0.980	1.265	0.246

Beta-diversity analysis of observed amplicon sequence variants (ASVs) considering different HM free glutamate concentrations for female and male infants. These data represent the comparison of β-diversity measures, including pseudo F-statistic and associated p-values, calculated using Bray-Curtis dissimilarity, Jaccard dissimilarity, weighted UniFrac distance, and unweighted UniFrac distance across human milk samples stratified by lactation stages (colostrum, transition, and mature) and gender (male vs. female).

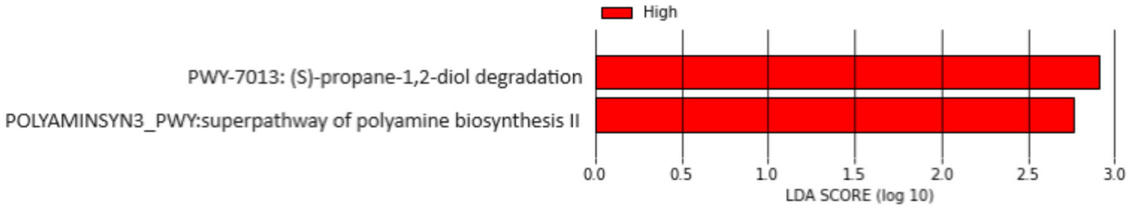

Figure S1. Linear discriminant analysis (LDA) scores, combined with effect size measurements (LEfSe) computed for differentially abundant predicted functions among three free glutamate concentrations in human milk samples. The length of the bar represents the log10 transformed LDA score. The threshold on the LDA score for discriminative pathways was set to ≥ 2.0.
